# DIGITAL MOBILITY MONITORING FOR HEALTHY AGING: RESULTS FROM LARGE AGING COHORTS IN EUROPE AND CANADA

**DOI:** 10.1093/geroni/igae098.0739

**Published:** 2024-12-31

**Authors:** Carl-Philipp Jansen, Marla Beauchamp

**Affiliations:** Chair; Discussant

## Abstract

Mobility has been deemed the ‘sixth vital sign’ because of its ability to predict critical health outcomes in later adulthood. Up to now, there is no consensus on the ideal measurement of mobility, but previous findings and experiences with movement sensors have led to regular application of digital technology. Important but unanswered questions remain around the potential of digital mobility outcomes (DMOs) to monitor individuals’ health status, to serve as markers for physical or functional decline, and to predict clinically meaningful events such as falls and hospitalisations. This could provide a better understanding of older adults’ and patients’ general health based on recurrent monitoring of their mobility. Against this background, data from major cohort studies in Europe and Canada are presented. Marla Beauchamp will present a recently proposed unified framework for mobility measurement, outlining three distinct aspects of mobility and investigating their relationship in a cohort of 1,200 older adults. Carl-Philipp Jansen will complement this data by looking at the same three aspects in a clinical cohort of 566 hip fracture patients, however, comparing their trajectories over one year. Laura Delgado-Ortiz will explore sensor-derived real-world walking in 550 older adults with COPD with healthy older counterparts, thereby capturing different characteristics of walking activity and gait from a single device worn at subjects’ lower back for one week. Karen Van Ooteghem will provide a look beyond single-sensor approaches, highlighting the large potential and presenting principles of multi-sensor approaches to provide comprehensive and meaningful information on mobility in older adults.

